# Profitability Analysis of Soybean Oil Processes

**DOI:** 10.3390/bioengineering4040083

**Published:** 2017-10-07

**Authors:** Ming-Hsun Cheng, Kurt A. Rosentrater

**Affiliations:** Department of Agricultural and Biosystems Engineering, Iowa State University, Ames, IA 50011, USA; minghsun@iastate.edu

**Keywords:** soybean oil, profitability, cash flow analysis, interest rate, net present value, payback time

## Abstract

Soybean oil production is the basic process for soybean applications. Cash flow analysis is used to estimate the profitability of a manufacturing venture. Besides capital investments, operating costs, and revenues, the interest rate is the factor to estimate the net present value (NPV), break-even points, and payback time; which are benchmarks for profitability evaluation. The positive NPV and reasonable payback time represent a profitable process, and provide an acceptable projection for real operating. Additionally, the capacity of the process is another critical factor. The extruding-expelling process and hexane extraction are the two typical approaches used in industry. When the capacities of annual oil production are larger than 12 and 173 million kg respectively, these two processes are profitable. The solvent free approach, known as enzyme assisted aqueous extraction process (EAEP), is profitable when the capacity is larger than 17 million kg of annual oil production.

## 1. Introduction

In the soybean refining process, extracting oil and concentrating protein contents are the two main purposes. The oil extraction is seen as the first step of soybean applications. The mechanical pressing technique, using pressure and heat to disrupt oleosome structure, was first used in the industry in the early 1940s [1]. However, low oil yield, oil darkening, and oil deterioration resulted from over-heating are the major disadvantages of mechanical pressing process. The extruding-expelling approach was introduced to mitigate the disadvantages and increase the oil recovery rate to over 70% from the typical mechanical pressing process [2]. According to the extruding-expelling process, an extrusion process replaces the heating process before soybean expelling which reduces the flaws resulting from over-heating.

Solvent extraction is another typical technique used in the industry, especially hexane extraction. For hexane extraction, the solubility of oil and hexane is the basic principal, and it can have over 99% of oil recovery rate [3]. The large amount of hexane used, 5~10:1 typical ratio of solvent to beans, leads to environmental and safety issues. The percolation extractor was invented to reduce the hexane usage and decrease the ratio of solvent to soybeans to about 1:1 [4]. However, the application of organic solvents still has been attracting concerns about safety and environmental impact problems.

An aqueous, solvent free extraction method, was introduced as an alternative in the 1950s [5]. The oil in water emulsion is formed after the extraction process due to the insolubility of water and oil, and the demulsification is applied to recover the oil from the emulsion. Based on the structure of oleosome stored in the cotyledons, proteases are commonly used in aqueous extraction to improve oil yield [6], and it is defined as enzymatic assisted aqueous extraction process (EAEP). In EAEP, a sufficient pretreatment, such as extrusion [7], ultrasonication [8], microwave heating [9], or ohmic heating [10], is required to break soybean tissue and produce porous soybean flakes which can improve oil recovery to over 80% [5].

For a manufacturing venture, the capital investment, operating costs, and revenues are the general indicators for evaluating the profit. Besides, the money sink throughout the service time of the project is another critical factor when estimating the profitability, and it can be performed by cash flow analysis. 

**Figure 1:**
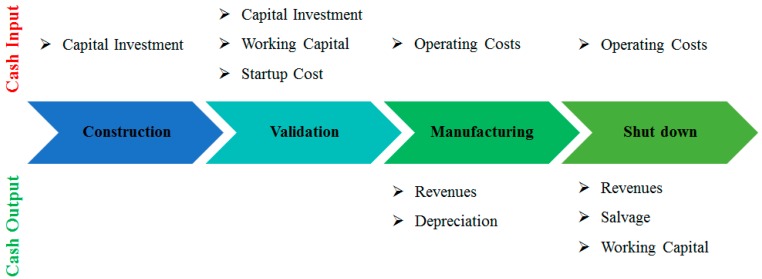
The schematic flow of cash flow analysis.

The concept of cash flow is illustrated in Figure 1. The venture can be divided into four periods: construction, validation, manufacturing, and shut-down. The capital investment is the basis for the construction period. In the validation period, working capital and startup cost are the cash inflow. As the process starts to operate, the operating costs are the cash inflow; while revenues and depreciation are defined as the profit. In the shut-down period, besides operating costs and revenues, the salvage of the capital investment and the working capital are compensated in the last year of the service time [11].

Time-value of money (TVM) is another factor for profit estimation. TVM means the value of money fluctuates with time and economic conditions. Therefore, it should be thought of as a commodity with time-depending value based on the interest rate [11,12]. The calculation is shown in Equation (1). C_t_ and C_0_ are the capital in the investment year (t) and the current year, respectively; i represents the interest rate.

(1) $$C_{t}=C_{0}{\left(1+i\right)}^{t}$$

Based on the revenues and profits earned from the process, the TVM also converts to a recent value for the profitability prediction. It is calculated by reversing Equation (1) to solve the C_0_, and it also can be expressed by a discount factor (f_d_) (Equation (2)). Also, the TVM is used to estimate the “Net Present Value” (NPV), which also estimates the profitability. NPV can be expressed in Equation (3) [13]. The positive NPV means the process is profitable and vice versa.

(2) $$C_{0}=\frac{C_{t}}{{\left(1+i\right)}^{t}}=C_{t}\times \frac{1}{{\left(1+i\right)}^{t}}=C_{t}\times f_{d}$$

(3) $$\mathrm{NPV}=\overset{t}{\underset{1}{\sum }}C_{t}f_{d}-\mathrm{Capital}\;\mathrm{Investment}$$

A similar concept is used for the internal rate of return (IRR) estimation. IRR is the indicator used to describe the interest rate at which the NPV is equal to 0. In other words, when the IRR of the process is larger than the interest rate (i) used for the profitability estimation, the process is profitable. Equation (3) is also used for the IRR calculation. In the calculation of IRR, NPV is replaced by 0, and the interest (i) can be solved as the IRR.

In soybean oil processes, the driving force of the extruding-expelling process, hexane extraction, and EAEP is the coproducts, soybean meal and insoluble fiber, and material cost basically which was performed by the sensitivity analyses from Cheng’s studies [14,15,16]. However, the profitability is less discussed in academic study. In this study, the cash flow analysis was used to evaluate the profitability of extruding-expelling, hexane extraction, and EAEP for soybean oil processes. 

## 2. Analysis Methods

### 2.1. General Assumptions

The assumptions are based on the TEA (techno-economic analysis) models built for extruding-expelling, hexane extraction, and EAEP [14,15,16]. The construction period is 30 months, with annual investments of 30%, 40%, and 30% of direct fixed cost (DFC) in the first three investment years individually. The startup period is 4 months; depreciation (DE) is 10 years with a straight-line method; and salvage (SV) is 5% of DFC. The basic interest (i) is 7% [12]. Also, the inputs and outputs data are based on 2015 prices. The plant is assumed to be built in Iowa, USA.

### 2.2. Data Inputs

In the profitability analysis of the soybean oil extraction process, the total capital investments of extruding-expelling process, hexane extraction, and EAEP are 26.6, 41.0, and 7.6 million dollars (2015 price) based on 30.8, 22.4, and 0.1 million kg of annual soybean oil production respectively. The different scales of production are also investigated, and the capital investments are estimated following 0.42, 0.34, and 0.35 power relationship of scaling up ratio for these three soybean oil extraction processes, respectively [14,15,16].

Operating costs generally include material costs, utility costs, and labor costs. The revenues are from soybean oil and other coproducts such as soybean hulls, soybean meal, insoluble fiber, etc. These inputs are listed in Table 1. For the extruding-expelling process and hexane extraction, the data are the average value from 2010–2015 based on 2015 price value. For EAEP estimation, the data is set as 2015 price because there is no plant using EAEP in the industry. The EAEP is proposed for combination with a corn-based ethanol production plant as an integrated soy-corn biorefinery system. The skim and insoluble fiber from EAEP are individually sold as a water supply and carbohydrate resource for corn-ethanol production.

### 2.3. Cash Flow Calculation

Based on the total capital investments, operating costs, and revenues estimations of these processes [4,5,6], cash flow analysis is performed following Table 2.

Gross profit (GP) and net profit (NP) are calculated first; with the net cash flow (NCF) obtained by adding capital investment (CI), GP, and NP. The cumulative cash flow (CCF) is the sum of the NCF from each year. CCF in the last year of plant service also represents the NPV without considering the interest (i). Additionally, the NPV with interest over 10% is also tested when the NPV is positive with the interest of 7%.

When the interest is considered, the discounted cash flow (DCF) is calculated by multiplying NCF with f_d_. The cumulative discounted cash flow (CDC) is obtained using the same methods as CCF calculations. The last year of CDC is the NPV of operating. Also, the IRR is obtained from SuperPro Designer v.9 model simulation.

## 3. Results and Discussions

### 3.1. Extruding-Expelling Process

**Figure 2:**
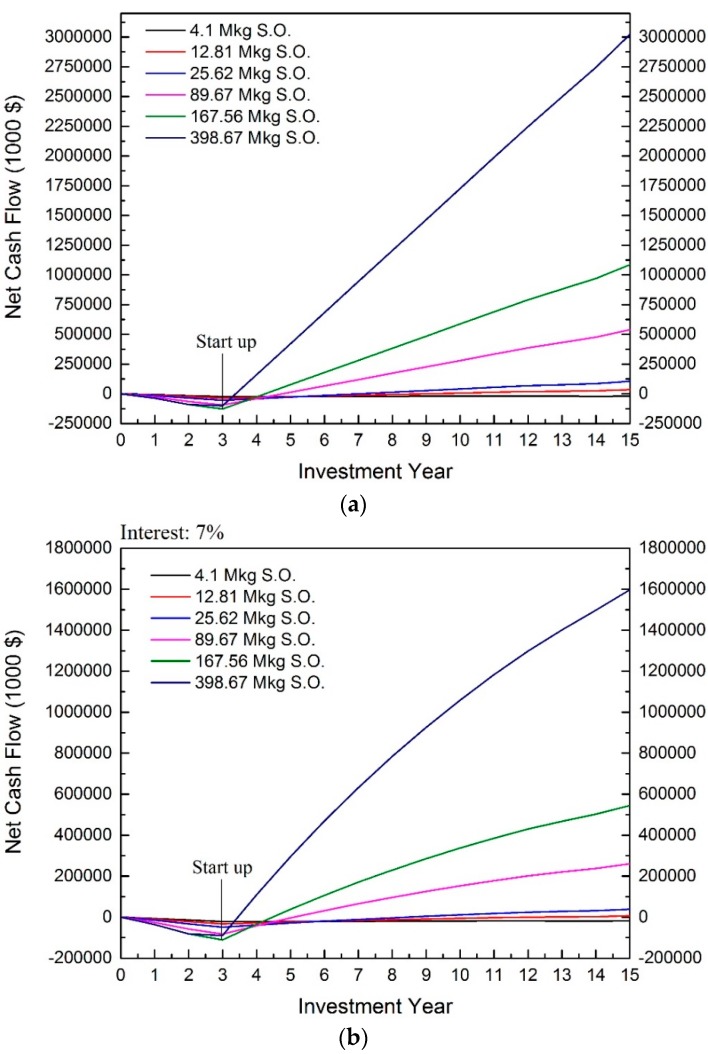
Cash flow of extruding-expelling process. (**a**) Interest rate is not considered; (**b**) 7% interest rate included. (S.O. indicates the annual soybean oil production).

The cash flow of the extruding-expelling process is shown in Figure 2. Results show the extruding-expelling process is profitable when the capacity is scaled up to 12.81 million kg of annual soybean oil production. The profitability is observed when interest rate is considered as well.

**Figure 3:**
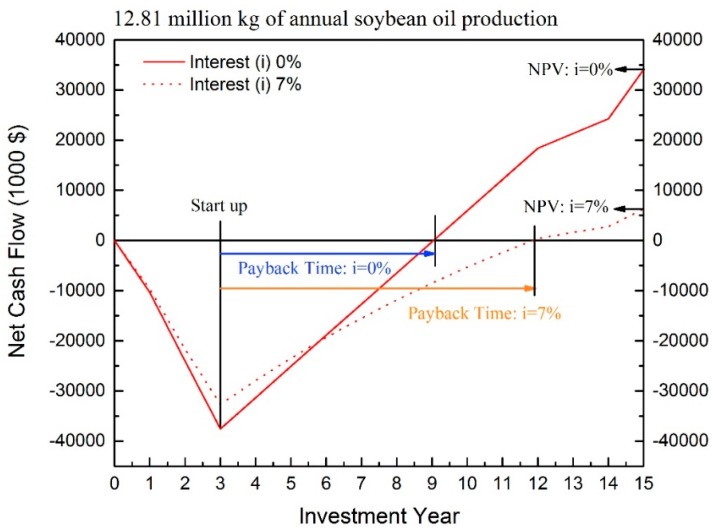
The profile of cash flow.

From Figure 2, the payback time and break-even point of the investment are also observed. The scale of 12.81 million kg of annual soybean oil production is used for further discussion (Figure 3). The cash outflow before the first three years demonstrates the total capital investment, mainly from DFC. After the third year, the cash inflow indicates that the process starts to earn revenues from products. And, the peak point is the startup time of the plant. The break-even point is found when the net cash flow meets 0. Therefore, the distance between startup time and the break-even point is the payback time of the process. The NPV is the net cash flow of the last year of plant service time. 

From Figure 2 and Figure 3, longer payback time and lower NPV are observed when the interest rate is considered. The longer payback time and lower NPV are expected when larger interest is applied. Additionally, the larger capacity result in shorter payback time and higher NPV. 

Based on the 7% interest rate, the NPVs of these six scales are $−18,319,000, $6,307,000, $38,263,000, $260,420,000, $544,552,000, and $1,597,494,000. Also, the IRR of the capacities larger than 4.1 million kg of annual oil production are larger than 7%, specifically 8.83%, 22.27%, 36.80%, 48.05%, and 93.83% respectively. When interest larger than 10% is considered, the positive NPVs are observed at the capacity of 25.26, 89.67, 167.56, and 398.6 million kg of annual soybean oil product, and the NPVs are $22,740, $192,960, $412,697, and $1,246,947 with interest of 10%, respectively. Conclusively, the capacities larger than 12 million kg of annual oil production are profitable when higher interest is considered.

### 3.2. Hexane Extraction

**Figure 4:**
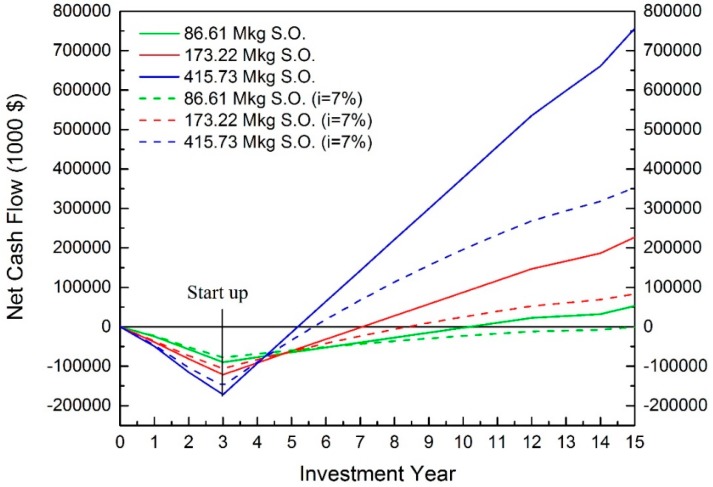
Cash flow of hexane extraction. (S.O. indicates the annual soybean oil production).

According to Figure 4, the trends of interest rate and capacity effects are similar to the extruding-expelling process. The larger capacity plants, without considering interest rate, result in higher NPV and shorter payback time. For the capacities of 173.22 and 415.73 million kg of annual oil production, positive NPVs are observed when the interest rate was included, and are $83,229,000 and $353,252,000, respectively. Their IRRs are 17.27% and 30.55% with payback times of about 2.5 and 3.5 years, respectively. Therefore, these two scales are profitable. 

The production scale of 86.61 million kg of annual oil production is estimated as a profitable process, with a positive net profit [5]. However, when the interest rate is considered, the NPV is $−283,000. It is risky to take this scale into operation. Based on the results of the simulation, the NPV of this scale is close to 0, indicating its IRR is close to 7% at 6.48%. 

When the higher interest, 10%, is considered, the positive NPVs are observed at the scales of 173.22 and 415.73 million kg of annual soybean oil productions which are $49,324 and $255,900 respectively. Therefore, capacities larger than 86 million kg of annual soybean oil production are profitable.

### 3.3. EAEP

**Figure 5:**
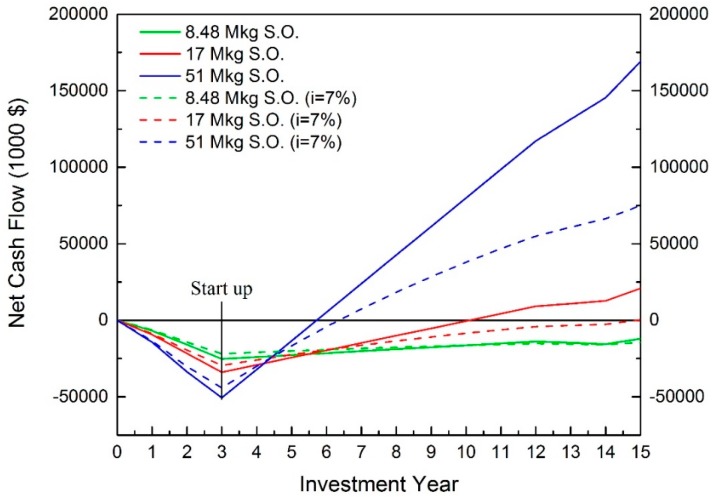
Cash flow of EAEP. (S.O. indicates the annual soybean oil production).

According to Figure 5, although the scale of 8.48 million kg of annual oil production has a positive net profit, negative NPVs are observed from both cases, with and without considering interest rate. This scale is not profitable in real operation.

For the scales of 17 and 51 million kg of annual oil production, positive NPVs are obtained. They are $20,808,000 and $169,114,000 without considering interest rate; and $307,000 and $74,957,000 when the interest rate is considered, respectively. Additionally, these two scales have reasonable payback times of about 3.5 and 12 years. 

**Figure 6:**
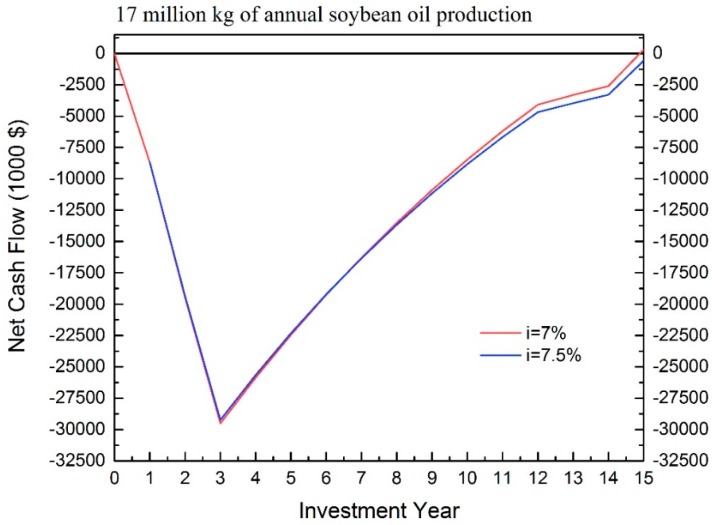
IRR prediction for EAEP.

For IRR, about 11% is achieved for the scale of 51 million kg of oil production. In the scale of 17 million kg of annual oil production, the IRR is about 7% because the NPV is close to 0. Figure 6 illustrates the approaching method for predicting the IRR. When a 7.5% interest rate was applied to the analysis, it resulted in a negative NPV. Therefore, the IRR allocates between 7–7.5%.

When interest of 10% is considered, the positive NPV can only obtained at the scale of 51 million kg of annual soybean oil production with $52,402. Therefore, the acceptable capacity of EAEP used in the industry needs to be larger than 17 million kg of annual soybean oil production. Additionally, the strategy of coproduct handling is also a critical factor for EAEP in industrial applications.

## 4. Conclusions

Based on cash flow analysis, the extruding-expelling process is profitable when the scale is over 4.1 million kg of annual oil production. For hexane extraction, the scales of 173.22 and 415.73 million kg of annual soybean oil production are profitable due to positive NPV at 7% and 10% interest rates. Though the positive NP and NPV without considering interest rate are obtained in the scale of 86.61 million kg of annual soybean oil production, the NPV becomes negative when the interest rate is included. This process is not profitable for real operating. As for EAEP, similar trends to those of hexane extraction are observed. The scale of 51 million kg of annual soybean oil production is accepted for industrial operation.

## Figures and Tables

**Table 1:** Operating costs and products selling price inputs (2015 price value).

				Extruding-Expelling	Hexane	EAEP *	Citation
Operating cost	Materials	Soybean	$/kg	0.44	0.44	0.35	[17]
		H_3_PO_4_	$/kg	0.60	0.60	N/A	[18]
		Water	$/L	0.001	0.001	0.001	[19]
		Hexane	$/kg	N/A	0.89	N/A	[15]
		NaOH	$/kg	N/A	N/A	20	[20]
		Protex 6L	$/kg	N/A	N/A	19.42	[16]
	Utility	Electricity	$/kwh	0.07	0.07	0.05	[21]
		Steam	$/MT	12	12	12	[18]
	Labor	Agricultural machine	$/hr	13.12	13.12	14.9	[22]
		Extraction	$/hr	13.12	20.86	22.49	[22]
		Hazardous material	$/hr	N/A	20.11	N/A	[22]
Revenues	Product	Soybean oil	$/kg	0.94	0.94	0.81	[17]
		Soybean hull	$/kg	N/A	0.21	0.21	[23]
		Soybean meal	$/kg	0.62	0.45	N/A	[17]
		Skim	$/L	N/A	N/A	0.01	[19]
		Insoluble fiber	$/kg	N/A	N/A	0.60	[16]

* Collected from 2015 price.

**Table 2:** The calculation of cash flow for soybean oil extraction investment.

Investment Year	Capital Investment (CI)	Operating Costs (OC)	Revenues (RS)	Gross Profit (GP)	Tax (T)	Depreciation (DE)	Net Profit (NP)	Net Cash Flow (NCF)	Cumulative Cash Flow (CCF)	Discount Factor (f_d_)	Discounted Cash Flow (DFC)	Cumulative Discounted Cash Flow (CDC)
1	30% DFC	0	0	OC-RS	0	0	GP -T+DE	CI+NP	∑NCF	(1/1.07)^t	NCF × f_d_	∑DFC
2	40% DFC	0	0	.	0	0	.	.	.	.	.	.
3	30% DFC + WC + SC	2 months OC	2 months RS	.	35% GP	0	.	.	.	.	.	.
4	0	OC	RS	.	.	90% DFC	.	.	.	.	.	.
5	.	.	.	.	.	.	.	.	.	.	.	.
13	.	.	.	.	.	0	.	.	.	.	.	.
14	.	.	.	.	.	0	.	.	.	.	.	.
15	WC + SV	.	.	.	.	0	.	.	.	.	.	NPV
